# High‐frequency oscillations in animal models of Alzheimer’s Disease

**DOI:** 10.1002/alz.089370

**Published:** 2025-01-09

**Authors:** Christos Lisgaras

**Affiliations:** ^1^ Nathan Kline Institute for Psychiatric Research, Orangeburg, NY, USA; NYU Langone Health, New York, NY USA

## Abstract

**Background:**

Clinical and preclinical evidence suggest that abnormal electrical activity strongly impacts outcomes in Alzheimer's disease (AD). Indeed, AD patients with interictal spikes (IIS) show faster cognitive decline than those without IIS. Furthermore, seizures in patients with AD have been suggested to accelerate disease progression. Here we asked whether high‐frequency oscillations (HFOs), which for the last 20 years have been considered a promising electrophysiological biomarker in epilepsy, also occur in preclinical AD models.

**Method:**

We used three mouse lines with AD features: Tg2576 mice, presenilin 2 knock‐out (PS2KO) mice, and the Ts65Dn model of Down's syndrome. We recorded HFOs using wideband (0.1–500 Hz, 2 kHz) intra‐hippocampal and cortical surface EEG at 1 month until 24 months of age during wakefulness, slow wave sleep (SWS), and rapid eye movement (REM) sleep. In addition, we analysed IIS and compared their occurrence with HFOs.

**Result:**

HFOs occurred in all transgenic mice but no controls. They were also detectable as early as 1 month of age and prior to amyloid beta plaque neuropathology. HFOs were most frequent during SWS sleep (vs REM sleep or wakefulness) when patients with AD also show abnormal EEG. Notably, HFOs were more frequent than IIS and occurred during more behavioral states. Last, high numbers of HFOs correlated with fewer IIS, suggesting for the first time possible competing dynamics among them in AD.

**Conclusion:**

Our data demonstrate that HFOs, an epilepsy biomarker with high translational value, occurs early during AD progression. Also, HFOs were the most abundant EEG abnormality when compared to IIS, and occurred in all behavioral states, suggesting they may become a promising surrogate marker of hyperexcitability in AD.

